# Herniation of the broad ligament… And the other side?

**DOI:** 10.1016/j.ijscr.2019.11.024

**Published:** 2019-11-19

**Authors:** J. Zemour, X. Coueffe, H. Fagot

**Affiliations:** Department of Visceral Surgery, CHU Réunion, Avenue du président Mitterrand, 97448 Saint Pierre, France

**Keywords:** Broad ligament, Internal hernia, Small bowel obstruction, Case report

## Abstract

**Introduction:**

Broad ligament herniation is a very uncommon cause of small bowel obstruction. Surgery permits reduction of the hernia and closure of the defect to prevent recurrence.

**Presentation of case:**

We report a rare case of a 35 year old woman admitted in our hospital for recurrence of a small-bowel obstruction, secondary to internal hernia. The patient underwent intestinal resection in 2018 for herniated left broad ligament of the uterus strangulated.

This time, the computed tomography revealed a double junctional syndrome with ileal dilation localized in right side of uterus. Emergency surgery confirmed internal hernia with passage of ileal loops through the right broad ligament. After reduction of the hernia, closure of the defect was performed to prevent recurrence. For the first time, a check of other classic abdominal hernias was carried out.

**Discussion:**

Internal hernias are responsible for less than 5% of mechanical bowel obstruction. Broad ligament hernias represent only 4–7% of these hernias.

The diagnosis is often delayed, because of its rarity and lack of surgical history. Surgery confirms the diagnosis, treats the occlusion and prevents recurrence.

The presence of several internal hernias has never been studied and no recommendation has been made on this topic.

**Conclusion:**

This case demonstrate the importance of a systematic exploration of other internal abdominal hernias during the surgery, especially a contralateral broad ligament hernia.

## Highlights

• Internal hernia is a classical cause of small bowel obstruction but remains rare.
• Discuss the appropriate management of internal hernia.
• Broad ligament hernias account for less than 5% of internal hernia.
• When performing surgery for broad ligament hernia, consider looking for contralateral hernia.

## 1 Introduction

Fifteen percent of all emergency department visits for acute abdominal pain are due to intestinal obstruction. The most common causes of intestinal obstruction include adhesions, neoplasms and incarcerated inguinal hernias [1,2]. The occurrence of abdominal internal hernias is rare, between 0,2%–0,9% of autopsies and 0,5–4,1% of cases of intestinal obstruction.

The Broad Ligament Herniation (BLH) is responsible of 4–7% of all cases of internal abdominal hernia [[3], [4], [5]].

The preoperative diagnosis is very difficult and abdominal Computed Tomography (CT) represents the diagnostic tool of choice. The surgery represents the last diagnostic tool and the first therapeutic modality [6].

The operative procedures are the reduction of the hernia and the closure of the defect to avoid the recurrence, but the research for other internal hernia is not classical proposed.

In this current case, we report a recurrence of small bowel obstruction due to BLH but on the other side.

This case has been reported in line with the SCARE criteria [7].

## 2 Presentation of case

We present a case of a 35 year old woman admitted through the emergency department of our hospital, for acute abdominal pain with nausea and vomiting. The symptoms started 4 h before the admission. She already underwent an intestinal resection in 2018 for herniated left broad ligament of the uterus strangulated.

Physical examination showed tachycardia, no fever and abdominal distension with diffuse tenderness, and no bowel sounds. The pain on palpation was diffuse and was similar to that which appears of last year.

Laboratory studies were normal with the exception of leukocytosis (withe blood cell count of 14,000/mm^3^).

**Fig. 1:**
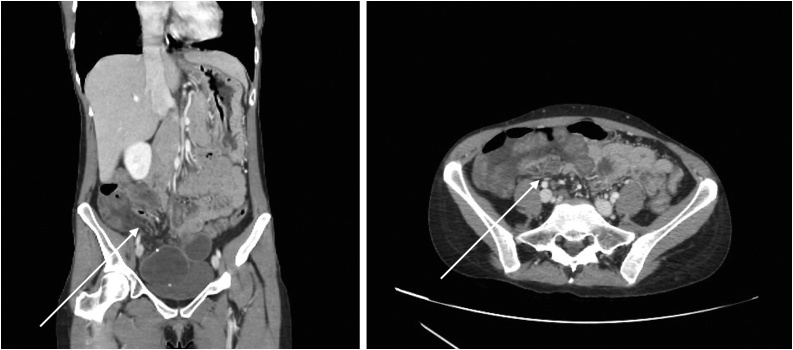
CT image reveals a closed dialed loop localized to the right of the uterus (arrow).

CT abdominal was performed because small bowel obstruction of a mechanical origin was suspected. The CT revealed a closed-loop dilatation of the ileum located in the pelvis close to the uterus due to an internal hernia through the right broad ligament (Fig. 1). A small amount of ascites and thickened intestinal walls were also noted.

We diagnosed a hernia through a defect in the right broad ligament with doubt on a digestive ischemia.

Initial management involved fluid therapy and nasogastric tube drainage.

**Fig. 2:**
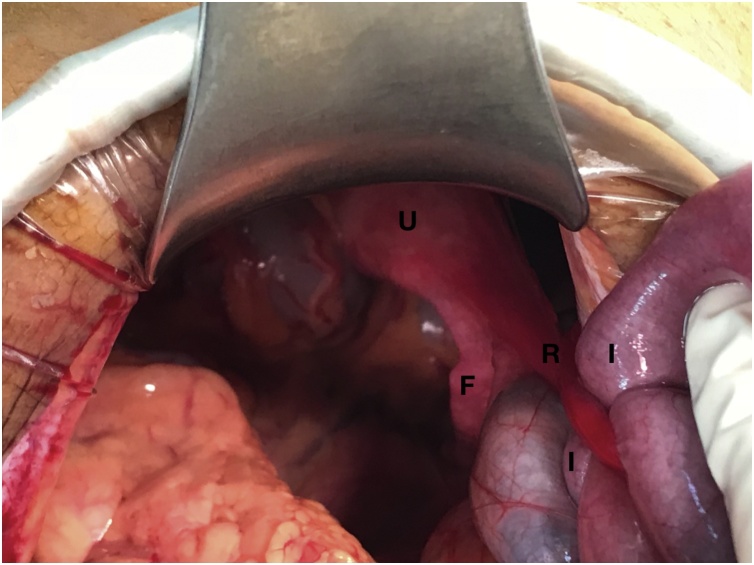
Intraoperative photograph. The herniated ileum loop can be observed through the broad ligament orifice. U: Uterus, F: Fallopian Tube, R: Round Ligament, I: Ileum.

We decided to perform an emergency exploratory laparotomy through midline incision because of history of abdominal surgery. It revealed a dilatation of small bowel loops and an internal hernia at the level of the right broad ligament that had a 2 cm defect with a 10 cm segment of terminal ileum incarcerated in the hernia (Fig. 2), which was completely liberated. The incarcerated small bowel was ischaemic, but motility and color were recovered after reduction, we did not perform resection.

The defect of the broad ligament was closed with a continuous suture to avoid recurrence.

A complete exploration was performed to check the integrity of the closure made on the left broad ligament and permitted to discover a small transmesenteric hernia. This hernia was closed by applying suture.

The postoperative course was excellent, with interruption of nausea, as well as abdominal distension. The patient tolerates oral feeding and returns to a normal intestinal transit. The patient was discharged fourth days after surgery. She had no complaints after 2 months of follow-up.

## 3 Discussion

Internal abdominal hernia is a very rare cause of small bowel obstruction, with an incidence of less than 5%. They are defined as the herniation of a hollow viscera by means of a natural or unnatural opening within the peritoneal cavity [8].

More than 50% of internal hernias reported in the literature are paraduodenal [9].

Less common types include pericaecal (13%), transmesenteric (8%), Winslow hiatal (8%), intersigmoidal (6%), supravesical and pelvic (6%) and transomental (4%) [10].

Internal hernias originating in broad ligament defects are very rare, comprising 4%–7% of all internal hernias. The first case was reported at autopsy by Quain in 1861 [11].

**Fig. 3:**
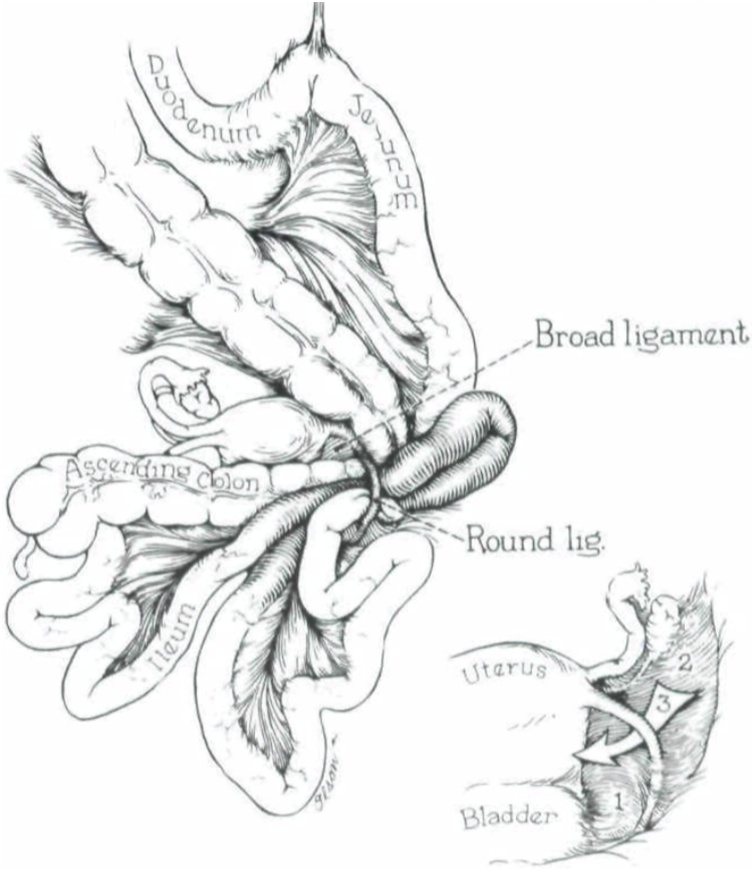
Illustration of the broad ligament; showing classification of broad ligament defects.

Cilley et al. [12] proposed an anatomical location based defect classification (Fig. 3): Type I defects occur throughout the entire broad ligament.

Type II, corresponds to a defect through the mesovarium and mesosalpynx above the round ligament.

Type III, which corresponds to a defect through the meso-ligamentum teres, between the round ligament and the remainder of the broad ligament.

Our patient had a complete fenestration through the broad ligament (type I), it is the most common type.

The cause of the defect in the broad ligament of the uterus may be congenital or acquired. Congenital cases have an embryologic explanation due to a developmental peritoneal defect around the uterus. In nulliparous patients, such defect may result from spontaneous rupture of cystic structures [13].

Acquired defects are secondary to surgical trauma, pregnancy and birth trauma, perforations following vaginal manipulation and inflammatory pelvic disease [14,15].

The defect of the broad ligament is often unilateral but can occasionally occur bilaterally, as in this case.

We did not find any case of the literature reporting a bilateral BLH complication.

It is normally considered as a severe condition due to the risk of strangulation and perforation of the hernial content. In most cases, the herniated viscera is the ileum, although hernia of the colon, ovary and ureter has been reported [16].

BLH are difficult to diagnose preoperatively, due to non-specific symptoms. The diagnosis is often uncertain, delayed due to the few cases and lack of surgical history. BLH is often revealed by small bowel obstruction but a delay in diagnosis may lead to strangulation and an increased risk of serious complications.

The preoperative diagnosis is very difficult but CT represents the best medical investigation, especially, multidetector CT with 3D reformatted images which provides significant advantages to evaluate the small bowel and surrounding structures, and shows its superiority to identify site, level and causes of small bowel obstruction including BLH.

Treatment is always surgical. The management of internal hernia through the broad ligament is two fold:

First, incarcerated contents reduction, and if necessary, non-viable bowel resection. Second, defect closure to prevent recurrent small bowel obstruction (using a suture or clip).

The laparoscopic approach is actually the treatment in the management of internal hernia for uncomplicated cases [17,18]. However, some factors are considered contraindicative to laparoscopic surgery like dilated small bowel >4 cm, bowel ischemia, history of severe adhesions and inflammatory bowel disease [17,19].

In our case, we preferred the laparotomy approach because the patient had an anterior surgery and bowel ischemia was suspected. This open surgery allowed a complete exploration, which is important due to the presence of other internal hernias.

The possibility of internal herniation through a defect in the broad ligament should be considered whenever a patient presents small bowel obstruction without obvious classical cause.

Because of a possible contralateral BHL, a systematic exploration on the other side must be carried out.

The surgery should be include the reduction hernia, the closure of defect and a complete abdominal exploration.

## 4 Conclusion

The occurrence of internal hernia through defect in the broad ligament is a very rare cause of small bowel obstruction.

The recurrence of small bowel obstruction on BLH but on the other side is exceptional, like in this case.

The surgeon, performing this procedure, should detect of possible other internal abdominal hernia, after the conventional treatment.

Systematic exploration of a contralateral BLH should be recommended.

## Funding

This study did not receive any funding.

## Ethical approval

Written informed consent was obtained from the patient for publication of this case report.

This case report is exempt from ethical approval by our institution.

## Consent

Informed consent was obtained from the patient

## Author contribution

J. Zemour: study concept, data collection, drafting of the paper and editing of the paper.

X. Coueffe: Literature review.

H. Fagot: Drafting of the paper.

## Registration of research studies

As this was a case report and not a clinical trial, this study does not require registration.

## Guarantor

Johanna Zemour is the guarantor for this study.

## Provenance and peer review

Not commissioned, externally peer-reviewed.

## Declaration of Competing Interest

The authors declare that they have no conflict of interest.
